# Better science with sex and gender: Facilitating the use of a sex and gender-based analysis in health research

**DOI:** 10.1186/1475-9276-8-14

**Published:** 2009-05-06

**Authors:** Joy L Johnson, Lorraine Greaves, Robin Repta

**Affiliations:** 1 NEXUS, School of Nursing, University of British Columbia, Vancouver, Canada; 2 British Columbia Centre of Excellence for Women's Health, Vancouver, Canada

## Abstract

Much work has been done to promote sex and gender-based analyses in health research and to think critically about the influence of sex and gender on health behaviours and outcomes. However, despite this increased attention on sex and gender, there remain obstacles to effectively applying and measuring these concepts in health research. Some health researchers continue to ignore the concepts of sex and gender or incorrectly conflate their meanings. We report on a primer that was developed by the authors to help researchers understand and use the concepts of sex and gender in their work. We provide detailed definitions of sex and gender, discuss a sex and gender-based analysis (SGBA), and suggest three approaches for incorporating sex and gender in health research at various stages of the research process. We discuss our knowledge translation process and share some of the challenges we faced in disseminating our primer with key stakeholders. In conclusion, we stress the need for continued attention to sex and gender in health research.

## Sex and gender in health research

In the context of doing more sensitive, precise and relevant health research, there is an increasing emphasis on attending to issues of sex and gender. Much work has been done to promote sex and gender-based analyses in health research and to think critically about the influence of sex and gender on health behaviours and outcomes [1-10]. This work is viewed as key to understanding and addressing health inequities that exist throughout the world. Several journals have published special issues in recent years, emphasizing the scientific, methodological, and ethical rationales for including sex and gender in health research [2,4,10,11]. Despite this increased attention on sex and gender, there remain obstacles to effectively applying these concepts in health research. Some health researchers continue to ignore the concepts of sex and gender or use the terms synonymously and thus incorrectly [9,12]. Certain disciplines are more familiar with these concepts than others; while gender has been a prominent concept in the social sciences for decades, and has therefore influenced social science health research, it has only relatively recently begun to enter the lexicon of biomedical and clinical health researchers. Thus, gender, which fundamentally refers to social and cultural influences, is often conflated or confused with sex, referring to the biological category of influences [9]. This conflation leads to confusion about the contributions of sex and gender to health, incomplete analysis and reporting in health research, and potential missed opportunities for developing appropriate medical interventions and policy responses [9].

To address these errors and omissions, researchers have begun to tackle the operational challenges of incorporating sex and gender in health research, providing methodological advice and realistic recommendations to researchers [8,9,11-14]. For example, Prins et al. [8] discuss the importance of developing methodologic standards for pharmacogenetic studies on sex and gender differences and address key issues around study design, analysis and result reporting. They provide an excellent checklist of issues to consider when studying the effect of sex in research, but do not differentiate between sex and gender, nor do they provide concrete ways to incorporate gender as a variable in health research. Phillips [13,14] on the other hand, addresses gender in health research, especially epidemiological approaches. She suggests the development of a proxy measure (or coefficient) for gender in women's health research, which could combine indicators of human rights, income, income distribution, and access to education and health care. Phillips acknowledges that this proxy measure of gender may not be relevant to men, and further development of the concept is required. Measurement techniques for addressing the effects of gender are crucial; however, Phillips' approach is somewhat confounding as she merges the related but fundamentally different concepts of sex and gender. The World Health Organization (WHO) provides a review of various gender tools, policies and guidelines designed to help measure the impact of gender on human health around the world [15]. This document identifies different layers of gender analysis, accounting for personal and community-level impacts of gender, and identifies questions to ask when investigating the interactions between sex and gender and their dual impact on health. A valuable resource, this document's strength is in its breadth and length, covering many NGO and aid organizations' gender policies. A condensed version of this 'tools' document, with more emphasis on incorporating sex and gender into every aspect of research design (particularly analysis) could better engage quantitative researchers and is needed. Thus, while these reviews, guidelines and suggestions for better use of the concepts of sex and gender in health research are valuable contributions, more comprehensive recommendations are needed for researchers to be prepared to use the concepts in all stages of the research process and in different fields and disciplines.

This paper builds on previous publications and responds to calls for additional guidelines on how to effectively incorporate sex and gender in health research [7-9]. We report on a primer that was developed by the authors to help researchers understand and use the concepts of sex and gender in their work [12]. The primer was published by the Women's Health Research Network (WHRN) in British Columbia, Canada in 2007 as a means of promoting sex and gender-based analyses in health research. The primer balanced some of the advanced theoretical discussions of sex and gender with workable suggestions for health researchers, in order to make the concepts more accessible. It was a practical starting point for health researchers across disciplines, involved in both human and animal research, who were beginning to use sex and gender in their research. The primer is available online at  and can be downloaded directly.

In this paper, we discuss the conceptual work that was the foundation for the primer, share the detailed definitions of sex and gender that we developed, and describe a three-prong approach to sex and gender-based analysis (SGBA). We review the practical suggestions that we offered for applying sex and gender in health research and share our experiences conducting 'knowledge translation' workshops as a means of promoting the primer to key stakeholders. In describing the challenges we faced in our knowledge translation process, we provide a case study on knee injuries that illustrates the benefit of applying SGBA to health research. Finally, we conclude by stressing the need for all health fields and disciplines to incorporate sex and gender as a matter of science and ethics.

## The case for using sex and gender

Many authors have written about the importance of using the concepts of sex and gender in health research [15-19]. The inclusion of sex and gender not only guarantees more comprehensive science, but can result in cost savings for the health care system, more effective policies and programs and is a matter of social justice [17]. For example, Aulakh and Anand discuss the importance of including sex and gender properly: previous research on stroke and aspirin wrongly led researchers to believe that aspirin was a useful preventative treatment for stroke in men only, and thousands of women likely missed this important therapy [18]. This emphasizes the ethical importance of accurately including sex and gender in health research, as omissions or the incorrect application of these concepts (e.g. errors in research design, analysis, reporting, etc. with respect to SGBA) can affect rates of morbidity and mortality [16-19]. Thus, it is critically important to understand and appreciate the impact of sex and gender, and attend to these concepts in health research correctly. However, the correct integration of sex and gender in research depends on consistent and clear definitions of the terms.

## Definitions

Using sex and gender accurately in health research requires a clear understanding of the two concepts because, as Krieger [9] confirms, "...our science will only be as clear and error-free as our thinking" (p. 656). While much has been written about these concepts, we found that definitions varied, particularly across disciplines. In order to provide clear recommendations of how to use these concepts, we first scanned the literature to assess how and where sex and gender are employed. Our initial scan produced a set of articles which we sorted into the following six categories: gender-based analysis and theories, policy and public health, tools/frameworks, interaction of gender and sex, masculinities/femininities, and examples from research. From here we looked at specific journals and reviewed the reference lists of these papers in order to obtain additional resources. This categorization system permitted us to generally review the issues and advances in conceptualizing sex and gender and helped us to identify gaps in knowledge and obstacles to implementing these concepts in research. Furthermore, our analytic process of assessing the field and reviewing the different usages and definitions of the terms underpinned our own definitions of sex and gender.

In surveying the quantitative literature, we found that gender is often mistakenly used as a substitute for sex; researchers claim 'gender differences' with respect to biology when they are in fact reporting differences according to sex. In the social sciences, where the distinction between sex and gender originated, the concepts are better understood but have evolved theoretically in ways that often seem to defy measurement. The concept of gender in particular has been thoroughly discussed and debated, with many definitions, sub-definitions, and theories offered [20,21]. However, transferring the latest theoretical developments into functional and operational models for health research and policy has yet to happen, so these important advances remain abstract and often unused in health research. To move forward, standardized definitions need to be accepted by all disciplines and amenable to both qualitative and quantitative research. We developed our definitions in order to incorporate the theoretical advancements in the social sciences in ways that basic scientists could appreciate and use. Our definitions below borrow from the definitions we developed in our primer and are referenced here with permission [12].

Sex is a multidimensional biological construct that encompasses anatomy, physiology, genes, and hormones, which together affect how we are labelled and treated in the world. Although conceptualizing sex usually relies on the female/male binary, in reality, individuals' sex characteristics exist on a fluid and medically or socially constructed continuum [22]. For example, research has revealed that while the "typical" sex chromosomes are XX for females and XY for males, there are many variations in this genetic chromosomal dichotomy, including XXY, XYY, XXX, and XO (no second chromosome). Therefore our common binary understanding of sex (male/female) is limiting and unrepresentative of the breadth and variety that exist with respect to human sex characteristics. Our common assumption that animals and humans are comprised of two sexes is reinforced by our limited language and has implications for research tools and design [23].

Sex has an enormous impact on human health in ways not previously understood [23]. For example, research has demonstrated that male and female bodies have innate physiological and hormonal differences that result in different responses to alcohol, drugs, and treatment [24]. In fact, the constitution of the typical female body has inherent differences when compared to the typical male body, from cellular metabolism to blood chemistry. Researchers now claim that "every organ in the body – not just those related to reproduction – has the capability to respond differently on the basis of sex" [[19], p. 935]. There are important sex-based differences at the cellular level arising from chromosomal dissimilarity. However, while we know that a male liver cell is not the same as a female liver cell, we do not know enough about the exact nature of these differences or whether these differences affect the development of disease or responses to treatment [16,17]. It is increasingly clear, therefore, that these various cellular differences can potentially create different patterns in the progression of disease in men and women and can lead to differences in health status and outcomes. There is a need to include both female and male animals and women and men in biomedical and clinical research in particular, because results from one group cannot be applied to the other [16-19]. Ignoring the influence of sex in research compromises the validity and generalizability of the findings and can be detrimental not just to the research enterprise but also to the health of individuals [19].

Gender is a multidimensional social construct that is culturally based and historically specific, and thus constantly changing. Gender refers to the socially prescribed and experienced dimensions of "femaleness" or "maleness" in a society, and is manifested at many levels [25]. The experience of gender is always linked to the social and political context. As such, gender is also intimately connected to social and economic status in systems where maleness is almost universally preferred over femaleness. The valuation of males over females is one way that "gender is a part of all human interactions" and "is a 'stable' form of structured inequality" [[24], p. 329]. While there is continued debate regarding the dimensions of gender, and its relationships to aspects of diversity, it is widely recognised that gendered experiences and cultural values often result in socially prescribed gender roles that dictate different behaviours, interests, expectations, and divisions of labour for women and men, girls and boys [26-28]. These gender roles are further reinforced by practices, processes and rules that affect gender identity at the individual level, gender relations at the interpersonal or group level, and institutional gender at a macro level [26].

Gender rolesreflect the behavioural norms applied to males and females in societies that influence their everyday actions, expectations, and experiences. They are expressed and enacted in a range of ways including dress codes, mannerisms, posture, and societal opinions of worthwhile contributions to make as a woman or a man. In some cultures, these roles are sharply defined and differentiated, allowing and disallowing women and men, girls and boys from certain tasks, jobs, opportunities, or spaces [22,23]. In other cultures, there is more gender equity and the lines between gender roles are more blurred. Either way, gender roles often categorize individuals and control behaviour within institutions such as the family, the labour force, or the educational system [26].

Gender identitydescribes how an individual sees themselves on the continua of female or male (or as a "third gender" or "two-spirited"), and influences their feelings and behaviours. All individuals develop their gender identity in the face of strong societal messages about the "correct" gender role for their presenting sex, but gender identities are malleable and actively constructed over time and culture, underpinning "an ongoing process of becoming" [[26], p. 309]. Gender identity is linked to social roles, aspirations, social interactions, behaviours, traits, characteristics, and body image and is influenced by prescribed gender roles and the extent to which individuals accept or resist them. Gender identity is evolving and not always stable. For example, an infant presenting with ambiguous genitalia is often assigned a gender by medical personnel, and then socialized accordingly [27]. Some individuals may experience disjunctions between their apparent sex and their identification with the other gender, leading to transgenderism, and sometimes desires for reassignment (surgical or otherwise). Finally, there are cultural differences that either allow or prohibit expressions of gender identity, such as the "hijra" in India who usually act in feminine ways, but who can be male or intersexed, though they are considered neither male nor female [28]. Growing up in a male or female body affects the gender identity individuals create/develop. For example, growing up female and being raised as members of a less desirable group can make it more difficult for girls to develop positive senses of themselves, which is required for good mental health [25].

Gender relations refer to how individuals interact with and are treated by others, based on their ascribed gender. Gender relations have a profound effect at all levels of society, and can restrict or open opportunities for individuals [29]. Gender relations interact with "race," ethnicity, class, ability, sexual orientation and other social locations and reflect differential power between women and men and between more or less powerful groups [28]. Gender relations affect personal relationships with others, and also guide interactions within social units, such as the family or the workplace. These relationships have a direct bearing on health [29]. For example, the gendered relationships between men and women have been found to influence the interpersonal dynamics related to tobacco reduction in pregnant and postpartum women [30]. Bottorff et al.'s 2006 study revealed that partner's expectations, support/pressure, and their personal tobacco routines influenced women's attempts to quit smoking during pregnancy and into the postpartum period [30]. Similarly, gendered and racialized relationships between workers and customers affect sales of tobacco to children [31,32]. DiFranzi et al. found higher incidences of tobacco sales to minors among male clerks [31] while Landrine et al. found that African-American and Latino children were asked about their age more often than White children when attempting to purchase cigarettes [32]. Furthermore, compliance with smoke free policies in bars has been found to be correlated with bartender gender, where patrons are more likely to comply when served by male staff [33]. The nature and details of these gendered interactions were not always explored; however, these examples illustrate the ways that gender operates relationally and in social contexts.

Institutionalized gender reflects the distribution of power between the genders in the political, educational, religious, media, medical, cultural and social institutions in any society. These powerful institutions shape the social norms that define, reproduce, and often justify different expectations and opportunities for women and men and girls and boys, such as social and family roles, job segregation, job limitations, dress codes, health practices, and differential access to resources such as money, food, or political power. These institutions often impose social controls through the ways that they organize, regulate, and uphold differential values for women and men [34]. These restrictions reinforce each other by relaying social processes of discrimination, inclusion and exclusion, creating cultural practices and traditions that are difficult to change and often come to be taken for granted. There are numerous examples of unequal and differential access for women and girls in particular, to resources that directly affect health and well-being. For example, girls are less likely than boys to be provided with health care, food, or education in many parts of the world [35]. Women are often malnourished due to the priority of feeding other family members first [36]. Even in developed countries, women are less likely than men to have an adequate income, and racialized women even less so, directly affecting their opportunity to achieve good health [34]. Thus, as Lorber and Farrell recognize, "Gender is built into the social order...The major social institutions of control – law, medicine, religion, politics – treat men and women differently" [[37], p. 1–2].

## Gender, Sex and Health Research

Gender and sex, while separate concepts, are inextricably linked and reciprocally influence each other. For example, a person's secondary sex characteristics (whether they have a penis or vagina or breasts) will influence how they are treated by others and will shape their life experiences (i.e., they will move through the world as a woman, man, intersexed or transsexual person). Gender also affects sex: men who view themselves as ultra-masculine and participate in high-risk sports and activities experience increases in their testosterone levels [38]. These examples illustrate the interconnectedness of sex and gender and help to explain why, in research, when people are asked to report their "sex" or their "gender" (when offered response categories "male" and "female") researchers are likely capturing both social and biological elements. For example, a person *may *report their gender based on both how they appear (secondary sex characteristics) and/or how they feel: masculine or feminine. It is this realization that likely led Prins et al. to suggest that "there is no difference in the use of the binary variables of sex and gender. The distinction between the two terms is usually relevant only when the mechanisms of influence are being studied" [[11], p. S107]. The binary variable "male" and "female" that is derived from most questionnaires and databases is useful for a beginning exploration of difference between males and females. Once established, we need to move beyond description and ask about whether the observed difference is caused by biological or social factors. It is in this exploration of the causal mechanism of difference and uniqueness where more refined definitions of sex and gender are required.

Sex and gender are multidimensional concepts, which means that any given individual is affected by multiple factors, including genetics, physiological characteristics, physical characteristics, gender identity, gender relations, and institutional gender. Additionally, sex and gender-related factors can interact and change as individuals move through the lifespan [3].

Given the complexity of understanding the effects of gender and health, what advice can be given to the researcher who wants to better incorporate and acknowledge the many issues related to sex and gender and health? While there are many ways to incorporate a sex and gender-based analysis (SGBA) in health research, in developing the primer we suggested three basic approaches in the hope that they would provide an entrée into a confusing field for a wider array of researchers. We deliberately wrote the primer in a simple manner in order to appeal to researchers who might not otherwise consider these concepts. Below we describe these approaches and reflect on our experience in trying to encourage others to use our suggestions. These approaches are meant to apply to all realms of health research: biomedical, clinical, health systems, social/cultural, and health policy, as well as all stages of the research process. It is important to recognize that integrating sex or gender into a study means more than simply adding men or women to a sample and instead requires changes throughout the research process.

For this reason, we developed three options to enable researchers to use SGBA at various stages of the research process. Option one involves revisiting an original study where data has already been collected and retroactively applying SGBA, reanalyzing or performing a secondary analysis. Option two helps researchers enhance an existing study with SGBA, making minor additions and changes to the research design. Option three encourages researchers to incorporate SGBA at the beginning of a study, and is therefore designed for projects that are able to make substantial changes or are still in the initial planning phase. We discuss these three options in detail below.

## Revisit an original study by applying SGBA, and or reanalyzing the data

The first of our three options is designed for research projects where data collection is complete, rendering a full-fledged SGBA difficult without additional and lengthy time investments. It is still possible to incorporate and account for sex and gender in these instances by critiquing and reanalyzing previously collected data. For example, researchers can disaggregate research results by sex, to explore whether differences exist, which is a necessary first step to engaging in sex and gender-sensitive research [39]. We do recognize that if sex was not a variable in the original data set, that reanalyzing by sex may not be possible. However, it is still possible to review and critique the way that sex and gender were used or omitted in a study, regardless of what specific data were collected. Researchers can critique and challenge the way that sex and gender were theorized, operationalized, and discussed in the literature review, and acknowledge if they were overlooked or confused [39]. Reanalyzing data by asking supplementary questions of previously collected data or further probing results in an attempt to explain sex or gender differences is constructive and can improve the applicability of research results. Performing a secondary analysis is another useful way of reanalyzing data that did not originally consider the concepts of sex and gender. A secondary analysis provides the opportunity to explore previously unexamined dimensions of the research, ask additional questions, compare data from other studies, or perform different statistical analyses [40]. Above all, as suggested by Eichler, asking the following questions of any work is always relevant and useful and can apply to any stage of the research process [39]. We paraphrase Eichler's questions below [39]:

1. Does the research question take one sex or gender as the norm, rather than stating explicitly who the research is applicable to? Make sure to avoid generalizing the findings to groups other than the one being studied.

2. Does the research question assume that women and men are uniform within their sex/gender groups? If so, consider that there are multiple differences between individuals of the same sex or gender and be mindful when reporting the findings to acknowledge the differences *among *groups of women or groups of men.

3. Revisit the literature review and examine how sex and gender are used in these studies. Are the terms *sex *and *gender *used accurately? How can your study present a more precise portrayal of sex and gender? If inaccuracies or omissions exist in the literature, make note of this in your own research to avoid perpetuating the confusion.

4. Are your measures for both sex and gender appropriate? If not, acknowledge this limitation and consider modifying your instruments if possible.

5. How were your data collected and how does this affect your results?

6. Does your analysis account for differences between the sexes and genders, and also within these groups? If not, reconsider how you can analyze the findings to account for these differences.

While research on the differences between the sexes is important and necessary, it is essential to move beyond the level of differences to explore how sex and gender operate in tandem to influence health outcomes and behaviours. Once differences between the sexes have been established, additional research is needed to explore whether sex, gender or both contribute to the differences. Krieger [9] provides an excellent table showing examples of the differential roles of gender relations and sex-linked biology on health outcomes. She identifies whether only gender, only sex-linked biology, neither, or both, are involved in the production of sex differences in case studies such as HIV/AIDS needle-stick injury among health care workers and parity among men and women with increased risk of melanoma [9]. This type of research illustrates the challenges of analysing sex and gender on several levels. For researchers new to the concept of gender, understanding and applying the many layers of gender can be a difficult task. A solution might be to begin with one layer of gender (e.g. gender identity or gender relations) in the analysis and move from there. Furthermore, as O'Brien et al. [41] have asserted, "Whilst the presentation of sex-disaggregated data (and explanations for apparent differences) is an important starting point for research on gender and health, it has the inherent danger of reifying differences between men and women, and homogeneity within gender classes" (p. 504). This danger is real and must be responded to with more reflection on within group differences and experiences and the social processes that affect them in achieving health. In this way, it is important to explain sex differences in ways that do not endorse stereotypes or use binary thinking, nor assume that all members of a sex or gender group experience risks to or opportunity for health equitably.

## Augment an existing research plan with SGBA

The second option is intended for research studies that are in the initial stages where data collection has not ended and where amendments and modifications are possible. This option encourages adding samples of men to a study on women, or samples of women to a study on men, to enable more rigorous and complete analyses. Dividing a sample by sex is also valuable, as this immediately contributes to more comprehensive findings than research on undifferentiated samples. Further dissecting samples by, or providing information about, age, ethnicity, socioeconomic status and other variables enables researchers to further investigate important health determinants.

Adding sex and gender-sensitive measures allows for deeper analysis of complexities in research. While adding a measure of sex or gender is usually only possible while data collection is still in progress, doing so can help to reveal and/or explain sex or gender differences, and also quantifies differences in ways that are often not possible otherwise. Measures that adopt a global perspective with respect to sex and gender can provide an additional advantageous lens. Examples of sex measures include anatomical measurements, like height, weight, and muscle mass, physiological measurements like sex hormones, and metabolism, and genetic sex chromosomes. Measures of gender include the Bem Sex Role Inventory [42], the Masculine Gender-Role Stress scale [43], and the Kobe Women's Health Indicators [44], to name just a few. All measures should be reviewed in light of recent theoretical and clinical progress made on sex and gender to ensure that measures are sensitive to the latest developments and accurately measuring the issue at hand (see our Option 1 checklist). Current measures may not be sufficient but are, for now, a means of addressing the issues. Acknowledging the limitations of a chosen measure is one way of circumventing this challenge. Additional work is needed to develop more precise and reflexive ways to operationalize and measure sex and gender.

Mixing qualitative and quantitative research methods can provide valuable considerations of sex and gender and can utilize the unique contributions that each approach offers. Supplementing a quantitative study with qualitative interviews or focus groups can further explain certain phenomena. Similarly, qualitative approaches can provide an initial examination of the issues to be further studied using quantitative methods. While not all studies need to use both qualitative and quantitative research methods, there are benefits to combining these two perspectives and can be a means of including sex and gender at a later stage of the research process.

The inclusion of female animals in preclinical research is crucial as every animal cell is also sexed [3,16], therefore excluding female animals has potentially extensive human health ramifications. The exclusion of female animals typically occurs as a means of controlling for hormonal variation. However, these hormonal variations are one of the key differences that deserve to be studied; therefore researchers need to learn to incorporate hormonal variations into study design to understand their important influence.

## Incorporate SGBA from the outset

Refocusing and reconceptualising a proposed study can incorporate concepts of sex and gender (and diversity) from the outset. As previously discussed, there is heterogeneity in relation to sex and gender within women [14,16]. For example, women have variance in their hormone levels and can have a range of gender identities. It is therefore important to incorporate concepts of gender and sex in studies that are relevant to women only (e.g., maternal mortality and reproductive health). Using SGBA in studies focused on women exclusively is not only possible but also important, as this type of study can examine issues of diversity among women (e.g., how race and socioeconomic status affect the health of women). Researching differences in susceptibility to diseases and conditions and responses to treatment among and between groups of women is a crucial dimension of single-sex studies that is often overlooked. These types of studies benefit from the application of SGBA models that are sensitive to issues of diversity. When beginning a study on women/females only, it is important to review the theoretical framework and methodology to confirm that they are appropriate for studying females, as research methodologies impact the type of questions that are asked, the process of data collection, and the analytical work that is done. Quantitative researchers need to be mindful of sample size when studying women only and take care not to over generalize their results to populations outside of the group(s) at hand.

Comparison studies can illuminate differences between and among groups of men and women, particularly with respect to variability in age, income, ability, socio-economic status, geography, ethnicity, etc. Using a longitudinal approach, or investigating trends over time, can uncover important gaps and differences. For example, research has shown that smoking rates among subgroups of men and women have changed over the last 20 years, and that specific groups of people are particularly vulnerable to tobacco addiction, including Aboriginal people, youth, and individuals with low-incomes [45]. These findings can provide insight and direction for future health policies and prevention efforts.

Multilevel studies make the simultaneous examination of multiple layers of sex and gender (and diversity) possible. Multilevel approaches, where the interplay and contributions of many sex, gender and diversity variables are studied, are becoming more important as ecological and other group-level health determinants are linked to individual factors [46]. Hierarchical linear models (HLM) are a multilevel approach that characterizes individuals and animals as nested within groups (e.g. according to sex, racial background, or occupation) as a means of investigating the interaction between individual-level and group-level variables [46]. This method is useful when exploring whether health outcomes for individuals or groups are correlated.

These three basic suggestions for improving health research were designed to appeal to new or established researchers, animal or human researchers, and those assessing already collected data or setting out to acquire new data. They provided some basic choices and suggestions in a field where new and established researchers alike often steer away from integrating considerations of sex and gender into health research because of disciplinary practices, a lack of will or a lack of understanding of the concepts and variables. While it is an emerging and constantly changing field where the concepts, theories and methods are constantly being improved and made more sophisticated, there is an urgent need to begin to involve more health researchers in the enterprise, utilizing basic approaches such as these.

## The Knowledge Translation Process

Knowledge translation (KT), defined as moving "knowledge to action" [[47], p. 22], has been identified as an important aspect of health research that is typically overlooked by researchers [47,48]. The importance of ensuring that research results are shared with stakeholders, particularly in health research, cannot be overstated: the consequences of ignoring this transfer of knowledge can range from less effective programming to increased morbidity and mortality in health research contexts [48]. With this in mind, there is increasing recognition among health researchers that KT must be incorporated into research projects from the outset and should involve users of research whenever possible.

As part of the knowledge translation strategy for the primer [12], the Women's Health Research Network (WHRN) organized and conducted workshops with researchers, students, clinicians and policy-makers across the province of British Columbia, Canada. The goal of these workshops was to introduce the primer and to break down the concepts of sex and gender so that participants would be able to understand the theoretical differences between sex and gender and recognize the importance and relevance of these concepts in their own research and programming. Above all, the workshops were meant to inspire participants to continue thinking about sex, gender and diversity in relation to health and to their own work.

The workshops assumed different forms, with some organized and led by the authors of the primer, and most coordinated and led by two staff members of the WHRN, Drs. Elana Brief and Colleen Reid. Workshops took place in various locations across the province of British Columbia, and were free of charge to participants and host institutions/organizations. At the time of writing this manuscript, 22 workshops have taken place since the primer was launched in April, 2007. Of these 22 workshops, 4 were for provincial health authorities, 12 were for academic departments at universities and colleges, and 6 were for government-funded research networks and research groups, all within British Columbia, Canada. The primer itself has been downloaded over 650 times by people all around the world who have found it useful for teaching, grant writing, and manuscript preparation purposes.

The workshops typically used a multi-method presentation format that involved a talk by the facilitators on the basic concepts of sex and gender, an illustrative power point presentation, activities to engage participants with the concepts, and both small and large group exercises to help participants incorporate sex and gender in their own work. Participants analyzed health issues like cardiovascular disease, tobacco use, and diabetes using sex and gender-based analyses in order to expand how they previously thought about these specific health issues. Participants were encouraged to create research strategies and approaches that could better account for sex and gender effects. These practical sessions provided hands-on training in the hopes that participants would be encouraged and prepared to use SGBA in the future. Facilitators found that kinaesthetic exercises were helpful, particularly in bringing the amorphous and fluid concept of gender to life. For example, one popular activity that facilitators used involved having participants construct a continuum of gender identities using pictures of well-known celebrities to illustrate pictorially the ideas of femininity and masculinity. With the image of the celebrity in mind, participants were asked to stand somewhere on the gender line-up between 100% masculine and 100% feminine. Participants then revealed their image and explained why they stood in that particular spot. Inevitably, explanations ranged from notions of gender identity, gender roles, and gender relations, to, in some instances, institutionalized gender. By moving gender from its complicated and abstract theoretical origins to a tangible "hands-on" and embodied group activity, participants were able to connect with the concept and became more comfortable with the idea of integrating gender into their work.

While we have enjoyed some success introducing these concepts to researchers across British Columbia, the process of sharing the primer with others has allowed us to appreciate how difficult it can be to incorporate sex and gender into research. Despite our best intentions to keep the format of the workshops simple and the concepts easy to understand, we often worried that participants hadn't fully grasped the concepts, or would be unable to integrate sex and gender into their work after they left the workshop.

One of the most difficult aspects of conducting the workshops was the limited measurement tools available to suggest to participants for use in their research and programs. Compounding this lack of resources was the varied level of experience with, and prior knowledge of, sex and gender. Thus, the facilitators were, ironically, often uncomfortable dealing with the issue of measurement directly, even though the measurement of sex and gender was a core concept within the primer. There is a dearth of appropriate and concrete tools to measure sex and gender, especially to suggest to an assorted mix of participants, so it was instead easier to provide a framework of key issues to consider and tailored case study examples for each group. Sharing the primer with others has illustrated to us the need for accurate and reflexive measures, particularly of gender, so that the field of gender and health can move forward and beyond the use of sex and gender as analytic tools. The development of concrete and accurate measures will also help to extend the conversation about sex and gender and health beyond those who are already familiar with these concepts. Concrete measures will better enable researchers to adopt SGBA.

Perhaps most useful in teaching about the health effects of sex and gender was the provision of case examples, which took participants through the process of applying SGBA and illustrated the impact of considering sex and gender in particular research instances. These examples were tailored to be specific to each audience so that while the concepts of sex and gender were new, the material was familiar and provided realistic examples of improvements that could be made. We provide a case study below to illustrate this method of KT that we found effective:

### Case Study: Knee Injury

Knee injuries are a curiously and sometimes controversially gendered phenomenon which, when investigated using a sex and gender-based analysis, reveal a number of important differences between men and women that have consequences for prevention, diagnosis, treatment, and patient care. Here, we illustrate how the application of SGBA could benefit studies related to knee injury at various stages of the research process, like in our 'three options' listed above:

#### 1. Revisit an original study by applying SGBA, reanalyzing or performing a secondary analysis

Sex-disaggregating previously collected data on anterior cruciate ligament (ACL) tears reveals that women are more likely than men to sustain an injury [49], more likely to report pain than men [50], and more likely to suffer from osteoarthritis [51]. Establishing that sex differences exist with respect to ACL injury should signal the importance of accounting for sex and gender in future ACL research, as the factors associated with ACL injury risk may not be the same for men and women. This difference would be missed without separating samples according to sex.

#### 2. Augment an existing research plan with SGBA

Using the knowledge that sex differences exist with respect to injury, researchers could attempt to explain this difference by examining different causal factors. By dividing a sample into men and women (or adding a sample of women), prospective studies examining risk factors for ACL injury in both men and women could focus on specific factors to see if and how they differ between the sexes and might contribute to injury. Researchers could also retrospectively compare injured individuals, male and female, to control cases to try and isolate specific factors that could place women at risk of injury. Studies such as these have confirmed many differences between men and women that could be causes of women's greater risk of ACL injury. For example, researchers have found that knee laxity, limb alignment, knee notch dimensions, and ligament size differ between the sexes and all have been theorized to cause women's greater risk of ACL injury [52]. Because of these anatomical differences, sex-specific knee replacements have been developed for men and women seeking knee replacements [53].

Adding a measure of hormonal influence in studies already designed to investigate casual factors would immediately provide more comprehensive findings, as hormonal factors are also believed to impact women's greater risk of injury [52].

Supplementing a quantitative study focused on the neuromuscular and biomechanical aspects of ACL injury with a qualitative investigation examining the impact of ACL injury in participants' daily lives, the process of negotiating treatment options or the experience of ACL repair could provide insight into this gendered subject. For example, qualitative research examining adherence to rehabilitation regimes has reported environmental, physical and psychological reasons that individuals are successful or unsuccessful in their rehabilitation attempts [54]. This type of qualitative research would benefit from a gender analysis that could consider the impact of gender roles on men and women's risk of injury and daily lives in ways that promote or impede rehabilitation (e.g. different work schedules, responsibilities at work and at home, and amount and type of physical exercise, etc).

#### 3. Incorporate SGBA from the outset

While it is now widely accepted that women are more prone to ACL tears than men due to the important research on sex differences in knee structure and function, research has not yet identified the specific mechanisms that lead to these sex differences. More research is needed to pinpoint the underlying factors that lead to discrepancies, and to document which factors lead to an increased risk for women in particular [49]. These types of studies will need to account for sex and gender from the outset. For example, studies focused specifically on women's ACL injuries have identified that sex hormones are likely one factor contributing to women's greater risk for ACL injury [55,56] and that women are more likely to suffer an injury during a particular phase of their menstrual cycles [55,56]. Research is still needed on the sex-based mechanisms that cause women's high rate of ACL injury so that prevention efforts can be developed.

Studies that incorporate SGBA from the outset are better equipped to investigate the impacts of gender. For example, Borkhoff et al. [57] examined gender at the institutional level and found that twice as many men than women were referred for total knee arthroplasty (knee replacement) in an Ontario-based study, despite similar symptoms and level of disability. This startling finding has led researchers to speculate that gender biases might be at play, where physicians consciously or unconsciously activated stereotypes about which gender is more likely to need total knee arthroplasty (TKA), and/or succeed with TKA [57]. Furthermore, there has been speculation that gender roles positively influence the way that men interact with physicians when seeking help for injured knees, and that women's narrative speaking style is not as effective in health care appointment settings as men's factual and to-the-point style [57].

These examples illustrate the benefit of utilizing a SBGA in health research and illustrate the 'value-added' of such an approach. Examples such as these proved helpful for the participants in our workshops and we hope that they are useful in this context as well as a means of understanding how SGBA might function. Above all, the process of SGBA means asking questions and thinking creatively about how aspects of sex and gender might influence the issue at hand. The workshops affirmed that more work is needed in this area, so that researchers are not only aware of sex and gender, but comfortable with the concepts and proficient in using them.

## Conclusion

The current landscape of sex, gender and health research will undoubtedly shift as more work is done in this area. We have suggested three main ways to use sex and gender in health research as a catalyst and starting point to help researchers think about these concepts in relation to their own work and interests. Introducing sex and gender in a comprehensive manner into health research heralds a new era, one that holds great promise for increasing our understanding of the origins of health and illness. We expect new developments in our understandings of sex and gender as research continues in these areas and more attention is paid to theoretical developments, questions of research design and the measurement issues related to sex and gender. We look forward to the articulation of more complex theories and measures of diversity, and growing insights into how all elements interact to produce health. Above all, recognizing sex and gender in health research is a necessity, in order to produce more accurate, rigorous, and valid results. Incorporating sex and gender into health research equals better science.

## Competing interests

The authors declare that they have no competing interests.

## Authors' contributions

JJ and LG developed the conceptual background and framework for the primer and paper. RR managed the project. JJ, LG and RR wrote and edited the paper.
